# Failure to Replicate the Association Between Fractional Anisotropy and the Serotonin Transporter Gene (5-HTTLPR, rs25531)

**DOI:** 10.3389/fnbeh.2018.00080

**Published:** 2018-04-30

**Authors:** Tim Klucken, Isabell Tapia León, Carlo Blecker, Onno Kruse, Tobias Stalder, Rudolf Stark

**Affiliations:** ^1^Department of Clinical Psychology, University of Siegen, Siegen, Germany; ^2^Bender Institute of Neuroimaging (BION), Justus Liebig University, Giessen, Germany; ^3^Department of Psychotherapy and Systems Neuroscience, Justus Liebig University, Giessen, Germany

**Keywords:** white matter microstructure integrity, diffusion tensor imaging, serotonin, connectivity, uncinate fasciculus

## Abstract

Recent work underlines the importance of alterations in white matter (e.g., measured by fractional anisotropy (FA)) as a neural vulnerability marker for psychiatric disorders. In this context, the uncinate fasciculus (UF), which connects the limbic system with prefrontal areas, has repeatedly been linked to psychiatric disorders, fear processing, and anxiety-related traits. Individual differences in FA may partly be genetically determined. Variation in the promoter region of the serotonin transporter gene (serotonin transporter-linked polymorphic region [5-HTTLPR]) is a particularly promising candidate in this context, which has been linked to psychiatric disorders as well as to limbic and prefrontal reactivity. However, findings on the association between the 5-HTTLPR and FA within the UF-tract have been heterogeneous. The present study investigated this relationship and extended previous work by considering different genetic classification approaches as well as sex effects in a human sample (*n* = 114). All participants were genotyped for the 5-HTTLPR and the rs25531 polymorphism. As a main result, we did not find any significant relationship between the 5-HTTLPR and FA in the UF-tract although power analyses showed an adequate power. In addition, genotype effects were neither found when different classification approaches were used nor when analyses were carried out in males or females only. The present findings suggest that the association of the 5-HTTLPR and FA seems to be a more labile phenomenon than previously assumed. Possible explanations and limitations are discussed.

## Introduction

The use of magnetic resonance imaging (MRI) has led to a deeper understanding of gene × brain interaction processes, which have been linked to the development, maintenance, and treatment of psychiatric disorders. In this context, a functional genetic variation within the promoter region of the serotonin transporter gene (SLC6A4), i.e., the serotonin transporter-linked polymorphic region (5-HTTLPR), has gained increasing attention over the last decade. A 43 base pair insertion/deletion located in the promoter region of the serotonin transporter gene results in a long (l-) and a short (s-) allele. Initial studies pointed to a (‘bi-allelic’) dominant effect with reduced 5-HTT availability and serotonergic functioning in s-allele carriers compared with homozygote l-allele carriers (Lesch et al., 1996; Stoltenberg et al., 2002; but Heinz et al., 2000). Currently, there is an ongoing debate about alternative (e.g., tri-allelic) classifications (Uher and McGuffin, 2008; Jonassen and Landrø, 2014) stimulated by findings that a single nucleotide polymorphism (A/G polymorphism [rs25531]) renders the L_G_-allele functionally equivalent to the s-alleles (i.e., reduced 5-HTT availability; “low-functioning group”; “SS, SL_G_, L_G_L_G_, SL_A_, L_A_L_G_”) in contrast to a “high-functioning group” (“L_A_L_A_”; Nakamura et al., 2000; Hu et al., 2006). Irrespectively of the classification approach, converging findings indicate an increased risk for the development of psychiatric disorders, like depression, in s-allele carriers and in the low-functioning group at least in interaction with stressful life events (Uher and McGuffin, 2008; Karg et al., 2011 but Risch et al., 2009). Furthermore, meta-analytic data indicate that homozygous s-allele carriers display increased cortisol stress reactivity (Miller et al., 2012). Regarding the interaction between 5-HTTLPR and neural reactivity, studies have repeatedly linked the s-allele (or the low-functioning group) to facilitated fear processing by showing increased blood oxygen level-dependent (BOLD) reactivity in the amygdala (Hariri et al., 2002, 2005; Bertolino et al., 2005; Canli et al., 2006; Munafò et al., 2008; Lonsdorf et al., 2011) and higher functional connectivity between the amygdala and the prefrontal cortex, which may reflect dysfunctional emotion regulation processes in this group (Alexander et al., 2009; Schardt et al., 2010; Lemogne et al., 2011; Kilpatrick et al., 2015; Klucken et al., 2015).

However, while the association between specific polymorphisms and functional connectivity has already been investigated extensively (Meyer-Lindenberg, 2009, 2010), structural connected (as measured be diffusion tensor imaging) has gained increased attention in the last years only (Alexander et al., 2007; Thomason and Thompson, 2011; Bracht et al., 2015; di Porzio, 2016). Diffusion tensor imaging offers a unique property to measure the coherence and direction of fiber tracts, which has been found to contribute to the development of psychiatric disorders and to be under genetic control (White et al., 2008; Kochunov et al., 2015).

In this context, the uncinated fasciculus (UF) is of special interest as a target white matter tract with respect to the 5-HTTLPR due to the following reasons: First, there is converging evidence that alterations in fractional anisotropy (FA) of the UF is associated with an increased vulnerability to psychiatric disorders as well as with neuroticism-related traits, although the exact relationship is under debate (Kim and Whalen, 2009; Montag et al., 2012). In detail, most (but not all) studies found decreased FA in the UF in patients with major depressive disorders or with anxiety disorders (see Ayling et al., 2012; Bracht et al., 2015; and White et al., 2008; for extensive reviews).

Second, the UF connects the limbic system with the prefrontal cortex (Ebeling and von Cramon, 1992). Structural and neural alterations in the limbic system and the prefrontal cortex are often linked to the 5-HTTLPR (e.g., Klucken et al., 2015). Third, from a functional perspective, it has been hypothesized that serotonergic transmission may be critical for neuroplasticity, which modulates the development and strength of connectivity of the PFC and limbic areas (e.g., the amygdala) involved in the serotonergic circuitry (Jonassen and Landrø, 2014). For instance, findings suggest that postnatal serotonergic transmission has long-term effects on the neuroplasticity within and around the amygdala and may thus alter its connectivity (Homberg et al., 2011). In addition, serotonin is involved in early differentiation and maturation of nerve cells not only in the amygdala but also in many other subcortical and cortical areas (Jonassen and Landrø, 2014). Finally, it has been shown that the UF-tract is also associated with fear-relevant bottom-up processes and altered emotion regulation, which are mainly processed by the limbic system and cortical structures (Montag et al., 2012; Modi et al., 2013; Zuurbier et al., 2013; Hermann et al., 2017) and are also closely related to the 5-HTTLPR (Lonsdorf et al., 2009, 2011; Lonsdorf and Kalisch, 2011).

To date, only three studies have investigated the relationship between the 5-HTTLPR and the UF-tract in healthy subjects with heterogeneous results: While one study showed decreased FA in the low-functional group (Pacheco et al., 2009), a second study showed increased FA in s-allele carriers, an effect which was found to interact with age (Jonassen et al., 2012). In another study, we also observed increased FA-values in s-allele carriers, but without any significant interactions with age (Klucken et al., 2015). However, it should be noted that the first two mentioned studies (Pacheco et al., 2009; Jonassen et al., 2012) were different from our previous study (Klucken et al., 2015), because they also focused on other research topics (e.g., age) and therefore analyzed a sample with a broader age range.

Given the current replication debate (Koole and Lakens, 2012; Stroebe and Strack, 2014; Strack, 2017), the aim of this study was to replicate and extend the previous findings by Klucken et al. (2015). The present study tries to investigate: (1) the association between the 5-HTTLPR and FA. In addition, (2) we sought to investigate effects for the bi-allelic and, as an aside, the tri-allelic classification approach separately in order to increase the depth of data and inform the current classification debate. Finally, (3) we also additionally analyzed 5-HTTLPR effects in females and males separately for identifying potential sex effects in DTI.

## Materials and Methods

### Subjects

One-hundred and fourteen participants (62 females; age_female_: *M* = 22.52 years; *SD* = 2.79 years; 52 males; age *M*_male_ = 24.48 years; *SD* = 3.29 years) took part in the study comprising an ethnically homogenous Caucasian sample. All participants were right-handed and had normal or corrected-to-normal vision. Participants were interviewed with a self-developed standardized interview for psychiatric disorders (e.g., Klucken et al., 2016). This short interview asks for the major disorders (e.g., mood and anxiety disorders, eating disorder, etc.) based on the Diagnostic and Statistical Manual of Mental Disorders (DSM)-IV Axis I diagnoses as well as traumata and the consumption of drugs. No subject had a history of any psychiatric or neurological treatment or a consumption of drugs. All participants were students of the University of Giessen and received either course credits or 10 € as a compensation. This study was carried out in accordance with the recommendations of the local ethics committee at the University of Giessen with written informed consent from all subjects. All subjects gave written informed consent in accordance with the Declaration of Helsinki. The protocol was approved by the local ethics committee at the University of Giessen.

### Power Analysis

*A priori* power analysis was conducted based on effect sizes reported in previous research in order to estimate the sample size necessary. The previously identified studies (Pacheco et al., 2009; Jonassen et al., 2012; Klucken et al., 2015) were used for the power analyses. The computed effect sizes between the 5-HTTLPR and FA-values in the UF-tract were used. A conservative threshold of 95% sensitivity (1-beta = 0.95) and a significance level of 0.05 were set for all power analyses. Given considerable variability in previously reported effect sizes, the estimates of necessary samples sizes varied between 26 and 98.

### Genotyping

DNA was extracted from buccal cells, using a standard commercial extraction kit (High Pure PCR Template Preparation Kit; Roche, Mannheim, Germany) in a MagNA Pure LC^®^ System (Roche). Subjects were genotyped for the 5-HTTLPR by means of polymerase chain reaction (PCR) and gel electrophoresis. DNA amplification reactions were performed as described below, using a Mastercycler^®^ ep (Eppendorf, Hamburg, Germany). Approximately 50 ng of genomic template DNA were amplified using the QIAGEN Multiplex PCR Master Mix (Qiagen, Germany) and 0.2 μM of each forward (5′-TCC TCC GCT TTG GCG CCT CTT CC-3′) and reverse (5′-TGG GGG TTG CAG GGG AGA TCC TG-3′) primer (TIB MOLBIOL, Berlin, Germany). Reactions were carried out in a total volume of 20 μl. Thermal cycling consisted of a 15 min initial denaturation phase at 95°C followed by 32 cycles of 94°C (30 s), 65, 5°C (90 s) and 72°C (60 s) each with a final extension step of 10 min at 72°C. To detect the rs25531, 9 μl of PCR products were digested by MspI in a 20 μl reaction assay containing 1× CutSmart-Puffer(BioLabs) at 37°C for 4 h. Finally, 16 μl of the restriction enzyme assay solution was separated by means of gel electrophoresis on a 1% agarose-gel in TBE (120 V, ca. 1:20 h) and visualized by Serva DNA Stain G.

The bi-allelic results (18 s/s-allele carriers (8 males), 61: s/l-allele carriers (24 males), 35 l/l-allele carriers (20 males) as well as results of the tri-allelic dichotomized model (*n* = 89 (35 males): “SS, SL_G_, L_G_L_G_, SL_A_, L_A_L_G_” vs. *n* = 25 (15 males): “L_A_L_A_”) are presented in the “Results” section.

### Diffusion Tensor Imaging

Imaging data were collected with a 3 Tesla whole-body tomograph (Siemens Prisma) and a 64-channel head coil. In addition to the DTI-sequence, a structural image acquisition was done and consisted of 176 T1-weighted sagittal slices (slice thickness = 0.94 mm; field of view (FoV) = 240 × 240 mm; TR = 1.58 s; TE = 2.3 s). Diffusion-weighted images were acquired using a single shot, pulsed gradient, EPI protocol (interleaved slice procedure; TR = 5.7 s; TE = 59 ms; FoV 232 mm × 232 mm; matrix size = 116 × 116, slice thickness: 2 mm, gap: 0.5 mm, voxel size: 2 mm × 2 mm × 2 mm, 64 directions, *b*-values = 0 and 1000 s/mm^2^). Skeletonization was carried out using the Tract-Based Spatial Statistics module implemented in FSL (Version 5.0.9). First, data were preprocessed (eddy current and head motion correction, brain mask for the DTI data, tensor calculation). Anisotropy was expressed as FA. The UF was taken from the Johns Hopkins University (JHU) DTI white matter atlas provided by the FSL software package (Wakana et al., 2007; Hua et al., 2008).

On the first-level, maximum FA were computed for each participant and each voxel and introduced as dependent variable for the group analyses. On the second level, group differences in FA were analyzed with respect to the bi-allelic approach and, as a supplement, on the tri-allelic approach.

Regarding the bi-allelic approach, two groups (s-allele group − l/l-allele group and vice versa) were compared (Klucken et al., 2015). Second, as a supplement, we also compared all three genotype groups with each other (i.e., s/s-allele group vs. l/l-allele group; s/s-allele group vs. s/l-allele group; s/l-allele group vs. l/l-allele group) to get more insight into potential genotype effects. Finally, we also explored an alternative approach comparing two groups with each other (s/s-allele carriers with l-allele carriers) due to heterogeneous classification strategies over studies.

In addition, we also explored the tri-allelic approach, comparing the low-functioning group (“SS, SL_G_, L_G_L_G_, SL_A_, L_A_L_G_”) with the high-functioning group (“L_A_L_A_”) to further explore differences based on alternative classification approaches. Further, we computed all above mentioned analyses for males and females separately, because prior studies indicate potential sex effects in context of DTI and/or 5-HTTLPR (Pacheco et al., 2009; Jonassen et al., 2012; Montag et al., 2012). Finally, we also tested for main effects of sex separately.

For all comparisons, *t*-statistics were computed and tested one-tailed using threshold-free cluster enhancement (TFCE; see Smith and Nichols, 2009) with permutation tests (program Randomize, version 2.9, FSL package). All reported *p*-values are FWE-corrected for the number of voxels under analysis. Significance level was set to α = 0.05.

## Results

The bi-allelic classification results in 18 s/s-allele carriers (8 males), 61 s/l-allele carriers (24 males) and 35 l/l-allele carriers (20 males). There was no significant deviation from Hardy–Weinberg Equilibrium [$X_{\text{(1)}}^{\text{2}}$ = 1.02; *p* = 0.31]. For the tri-allelic dichotomized model, the low-functioning group (“SS, SL_G_, L_G_L_G_, SL_A_, L_A_L_G_”) included 89 participants (35 males) and 25 (15 males) in the high-functioning group (“L_A_L_A_”). There were no significant differences with respect to age (*F*_(2,113)_ = 2.45; *p* = 0.78) and sex ($X_{\text{(2)}}^{\text{2}}$ ≥ 2.85; *p* = 0.24) in the bi-allelic (see Table 1) and in the tri-allelic classification approach (sex: $X_{\text{(1)}}^{\text{2}}$ = 2.49; *p* = 0.082; age: *T*_(112)_ = 0.378; *p* = 0.71).

**Table 1:** Demographics (age, sex) characteristics with means and standard deviations and significance of group differences for the three genotype groups.

	s/s	s/l	l/l	*p*
Age	23.22 (3.2)	23.61 (3.4)	23.17 (2.8)	0.783
Age range	19–29	18–32	19–30
Sex (female/male)	10/8	37/24	15/20	0.240

### Bi-Allelic Classification

In the first analysis, we compared the s-allele group with the l/l-allele group (see Table 2). We did not find any significant increased FA in any parts of the UF-tract in the s-allele group as compared to the l/l-allele group in the left (*p* = 0.65) and the right UF-tract (*p* = 0.14). In addition, no significantly increased FA was observed in the l/l-allele group as compared to the s-allele group (left UF: *p* = 51; right UF-tract: *p* = 0.28). Further detailed group analyses (i.e., s/s-allele group − l/l-allele group) also provided no evidence for any significant association between the 5-HTTLPR and altered FA-values in the left (*p* = 0.70) or in the right (*p* = 0.22) UF-tract. We also could not find any significantly increased FA-values in the l/l-allele group as compared to the s/s-allele group (left UF-tract: *p* = 0.35; right UF-tract: *p* = 0.21).

**Table 2:** Analyses of fractional anisotropy (FA) in the uncinate fasciculus (UF) with respect to the main results of this study (bi-allelic classification approach) using one-tailed *t*-tests.

Analyses	Side	*T*_max_	*p*_FWE–corr_
s-allele group −	L	1.26	0.65
l/l allele group	R	2.42	0.14
l/l allele group −	L	1.53	0.51
s-allele group	R	2.15	0.28
s/s-allele group −	L	1.03	0.70
l/l-allele group	R	2.17	0.22
l/l-allele group −	L	1.70	0.35
s/s-allele group	R	2.17	0.21

*L, left side; R, right side; FWW, family-wise error*.

Sex-specific analyses were conducted to analyze if 5-HTTLPR group differences emerged in males or in females. However, no increased FA could be reported in the s-allele group as compared to the l/l-allele group in males (left UF-tract: *p* = 0.65; right UF-tract: *p* = 0.18), or in females (left UF-tract: *p* = 0.56; right UF-tract: *p* = 0.20). In addition, no increased FA-values were observed in the s/s-allele group compared with the l/l allele group in males (left UF-tract: *p* = 0.66; right UF-tract: *p* = 0.27) and in females (left UF-tract: *p* = 0.10; right UF-tract: *p* = 0.54).

### Tri-Allelic Classification

As an aside, we also compared the low-functioning group with the high-functioning group. However, irrespectively of the classification approach, we did not find any significantly increased FA in the UF-tract in the low compared with the high-functional group (left UF-tract: *p* = 0.64; right UF-tract: *p* = 0.10) or vice versa (left UF-tract: *p* = 0.58; right UF-tract: *p* = 0.33). We analyzed potential genotype effects in males and females separately. In males, we did not observed any increased FA-values in the low-functional group as compared with the high-functioning group (left UF-tract: *p* = 0.70; right UF-tract: *p* = 0.10) or vice versa (left UF-tract: *p* = 0.46; right UF-tract: *p* = 0.57). Similarly, no effects could be found in the female group comparing the low-functioning group with the high-functioning group (left UF-tract: *p* = 0.68; right UF-tract: *p* = 0.55) or in the opposite direction (left UF-tract: *p* = 0.33; right UF-tract: *p* = 0.52).

## Discussion

The present study tried to replicate the FA-results by Klucken et al. (2015) and explored the association between alterations in FA within the UF and the 5-HTTLPR in a healthy human sample comprising males and females. To enhance the interpretability of findings, we computed the impact of the 5-HTTLPR with different classification approaches as well as sex-specific effects on FA-values. The results showed no significant association between the 5-HTTLPR and alterations in FA-values within the UF. In addition, results did not change significantly using different classification approaches for the 5-HTTLPR. Finally, no specific 5-HTTLPR effects could be observed in separate analyses in males or females.

In sum, the present findings could not replicate previous results of an association between the 5-HTTLPR and alterations in FA-values in the UF. We neither found increased nor decreased FA-values in l-allele carriers, which have been reported in prior studies (Pacheco et al., 2009; Jonassen and Landrø, 2014; Klucken et al., 2015). In order to explain these inconsistent findings, different (*post hoc*) explanations and potential limitations need to be considered.

It is important to note that alterations in FA measured by diffusion tensor imaging may be driven by different structural facets, like variations in intra-voxel orientational dispersion, myelination, packing density, membrane permeability, or axon diameters (Jones et al., 2013). Thus, it is conceivable that only some of these aspects might be influenced by serotonergic transmission, which may reduce the actual strength of this gene × brain association. Correspondingly, several authors have suggested to avoid the term ”white matter microstructure integrity” since alterations in FA-values are not necessarily based on integrity (Jones et al., 2013).

In addition to the mentioned biological considerations, methodological differences may have influenced the results. Previous studies used smaller sample sizes and/or females only. Moreover, the present study used a different DTI-sequence in contrast to previous studies. These methodological differences may impact accuracy and signal-to-noise ratio. For instance, some authors argued that Diffusion-weighted imaging with >20 directions are more likely to accurately quantify the white matter structure and the FA-values by improving the signal-to-noise ratio (Giannelli et al., 2009). In the current study, a DTI sequence that encodes for 64 directions of diffusion was used in contrast to the previous study (Klucken et al., 2015), which could in turn be more sensitive to detecting alterations in FA providing greater confidence in the results. In addition, there is evidence that inflammation and other processes may impact FA-analyses irrespectively of other biological and methodological aspects. Moreover, it should be noted that the present study tried to replicate our previous results (Klucken et al., 2015) and differed in the DTI sequence and the statistical analyses to the two other previous studies (Pacheco et al., 2009; Jonassen et al., 2012). For instance, Jonassen et al. (2012) investigated age-specific effects by exploring a broad age range, while the present study explored a student sample (age range: 18–32 years). Pacheco et al. (2009) statistically “controlled for” differences based on ethnicity, while the present study investigated Caucasian participants with a European background only. Pacheco et al. (2009) also used a different statistical approach and conducted regression analyses by entering the number of low-expressing alleles as variable, while the present study conducted group analyses to ensure comparability to our previous study (Klucken et al., 2015), which investigated associations of the 5-HTTLPR, brain activations, and structural differences.

It is also conceivable that the association between structural coupling and genetic variations is more pronounced in interaction with stressful conditions, like the development of psychiatric disorders or stressful life events. In line with this notion, a series of studies found no main effects but (only) interaction effects between FA-values and 5-HTTLPR on specific psychiatric symptoms and treatment responses for different tracts and psychiatric disorders (Tatham et al., 2016, 2017). In congruence with this, Kelly et al. (2014) also observed similar findings for further genotypes by showing no association between specific different genetic variations (e.g., for schizophrenia) and white matter alterations in healthy subjects, which already has been confirmed in meta-analyses for patients (Ellison-Wright and Bullmore, 2009).

However, while initial studies reported an association between altered FA-values and psychiatric disorders, emotional processing, or genetic variations, current studies have seriously challenged these findings. Recent studies observed no associations between white matter FA-values and psychiatric disorders (Olvet et al., 2016; Delaparte et al., 2017) or even with different polygenic risk factors in a large (UK Biobank) sample (Reus et al., 2017). In addition, it should be noted that our results are limited to the studied age range, while other studies explored samples with a broader age range (Jonassen and Landrø, 2014). The small number of participants for analyses on sex-specific differences in genotype groups is also likely to decrease the power of respective tests. Finally, as a limitation, no toxicological analyses were conducted. Further studies should investigate such potential differences in more detail.

In sum, we would like to point out that the present results do not principally argue against gene × (structural) brain interactions. The surprising (null) results may also support the view that the reported effects may exist in specific constellations only, or are relatively small, and/or may require specific parameters, which are not entirely clear to date. However, it is also possible that genome wide association studies are more promising than (single) candidate gene analyses in context of functional and structural alterations as well as diffusion tensor imaging.

## Author Contributions

TK, ITL, CB, OK and RS designed the experiment. TK, ITL and OK collected the data. TK, ITL, CB, OK, TS and RS were involved in data analyses and the interpretation of data for the work and wrote the manuscript.

## Conflict of Interest Statement

The authors declare that the research was conducted in the absence of any commercial or financial relationships that could be construed as a potential conflict of interest.
